# Diagnostic Value of Delayed Contrast-Enhanced Cardiac Computed Tomography for Detecting Left Atrial Appendage Thrombus in Patients With Atrial Fibrillation

**DOI:** 10.3389/fcvm.2022.847163

**Published:** 2022-04-28

**Authors:** Xiang-Nan Li, Jing-Xi Wang, Qing Wei, Xian-Bo Yu, Yu-Tao Zhou, Xiao-Yan Ma, Na Zhao, Bin Lu

**Affiliations:** ^1^State Key Laboratory of Cardiovascular Disease, Department of Radiology, National Center for Cardiovascular Diseases, Fuwai Hospital, Chinese Academy of Medical Sciences and Peking Union Medical College, Beijing, China; ^2^State Key Laboratory of Cardiovascular Disease, Department of Ultrasonography, National Center for Cardiovascular Diseases, Fuwai Hospital, Chinese Academy of Medical Sciences and Peking Union Medical College, Beijing, China; ^3^CT Collaboration, Siemens Healthineers Ltd., Beijing, China

**Keywords:** computed tomography angiography, atrial fibrillation, thrombosis, echocardiography, transesophageal

## Abstract

**Objective:**

Delayed enhancement cardiac CT is a reliable tool for the diagnosis of left atrial appendage thrombus but limited for scanning heterogeneity. We aimed to explore the improvement of the 1 and 3-min delay phase at the diagnostic level to detect left atrial appendage thrombus, in order to set up a reasonable CT scanning scheme.

**Materials and Methods:**

A total of 6,524 patients were continuously retrieved from January 2015 to September 2020 retrospectively. The patients had undergone Transesophageal echocardiography (TEE) and cardiac CT with complete period include the arterial enhancement phase, 1 and 3-min delay phase, TEE were used as the reference standard. The final study included 329 patients. Three experienced radiologists independently assessed each phase of the cardiac CT images for thrombus diagnosis. We explored the improvement of the diagnostic ability of different delayed contrast-enhanced phases for left atrial appendage thrombus detection. Multiple logistic regression analysis were used for further high-risk stratification to avoid an additional 1-min delayed scan.

**Results:**

In total, 29 thrombosis were detected at TEE. For all cardiac CT phases, sensitivity and negative predictive were 100%. The specificity were 0.54, 0.93, and 1.00, respectively; The positive predictive values (PPV) were 0.17, 0.57, and 1.00, respectively; Area under curve (AUC) were 0.75, 0.95, and 0.98, respectively. High risk factors that cannot be clearly diagnosed with 1-min delay phase included reduced cardiac function, increased CHA2DS2-VAScscore and left atrial enlargement. Compared with the arterial enhanced phase, increased radiation doses in the 1 and 3-min delay phases were 1.7 ± 1.3 msv and 1.5 ± 0.8 msv (mean ± standard deviation).

**Conclusion:**

Using TEE as the reference standard, early contrast-enhanced CT scanning with 1 and 3-min delay is necessary for the diagnosis of left appendage thrombus, which could significantly improve the diagnostic efficiency. Patients with high-risk stratification are suitable for direct 3-min delayed scanning.

## Introduction

Atrial fibrillation (AF) is one of the most common arrhythmias which has become an increasingly serious health care problem for elderly populations (1, 2). Left atrial appendage, the most prone site of cardiogenic thrombus, is specifically implicated in patients with atrial fibrillation. Thus, timely and accurate evaluation of left atrial appendage thrombus has become a necessary clinical demand. The 2020 atrial fibrillation management guidelines clearly proposed the use of Transesophageal echocardiography (TEE) to replace the conventional 3-week anticoagulant therapy before radiofrequency ablation (3). TEE is considered as the gold standard method for detecting left atrial appendage thrombus, with sensitivity nearing 100% and specificity close to 99% (4–6). However, TEE is a semi-invasive test that usually requires the use of sedatives.

Cardiac computed tomography (CT) has recently been proved as a reliable, established, and widely used technique for the detection of cardiac thrombus (7–11). At present, cardiac CT has better spatial resolution than TEE and can further reduce the number of images generated by a patient’s respiratory coordination (12–14). However, cardiac CT was not recommended to be written into the guidelines. A jointly published expert consensus in 2017 pointed out that this was due to the heterogeneity of cardiac CT scanning schemes and lacking of the high evidence-based level research (15).

Although cardiac CT has a highly sensitive ability to exclude cardiac thrombus, early enhanced imaging cannot intuitively distinguish blood circulation stasis and thrombus in the left atrial appendage or other cardiac parts. The consequence of this is a low specificity and positive predictive value through the use of single-phase enhanced scanning alone (16, 17). Previous studies have shown that additional delayed scanning can improve diagnostic accuracy (18–20). However, the reported delay time ranges from 30 s to 3 min, resulting in differences between the reported negative predictive value and the positive predictive value. This ultimately hinders the clinical practice of using cardiac CT to diagnose left atrial appendage thrombus, and clinicians are prone to confusion when reading the diagnostic report, could resulting in excessive thrombolytic and anticoagulant strategies.

Therefore, through the continuous collection of the cardiac CT scans for 5 years, we summarized the cardiac CT scanning scheme, image quality, and radiation dose. Additionally, we studied the role of early delayed scanning in improving diagnostic ability with TEE as the reference standard.

## Materials and Methods

### Study Population

**FIGURE 1:**
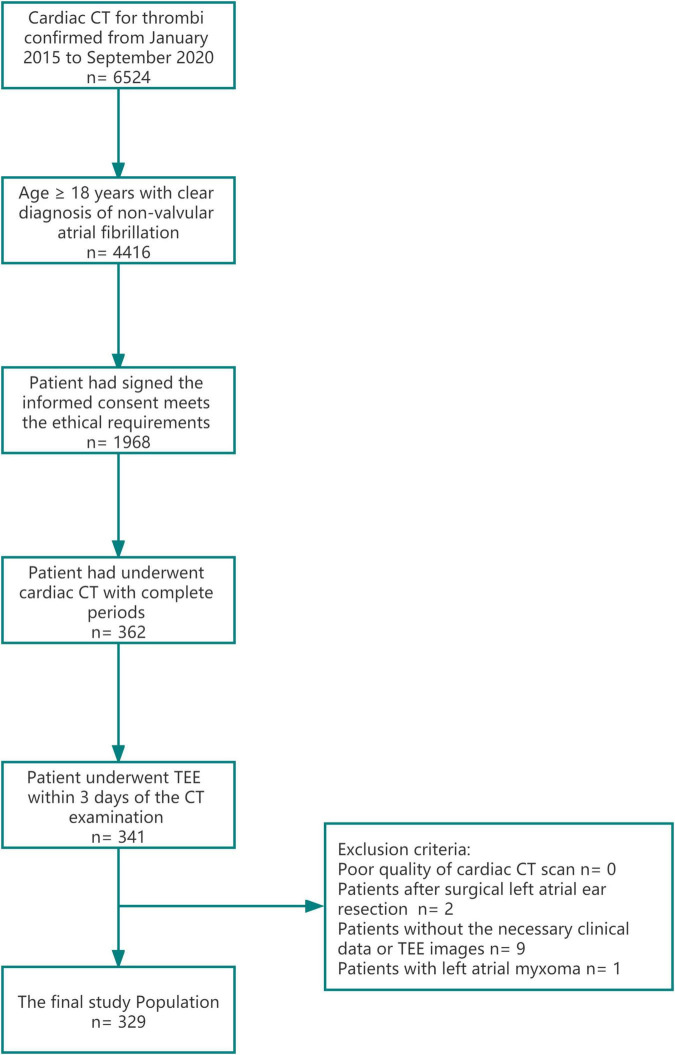
A study flow-chart of patients inclusion and exclusion. Cardiac CT with complete period include the arterial enhancement phase, 1 and 3-min delay phase.

Our institutional review board approved this retrospective study and informed consent was waived for meeting the exemption standard. The current study was a single-center retrospective study that included 5-years consecutive patients with atrial fibrillation from January 2015 to December 2020 in our central hospital. The inclusion and exclusion criteria of patients were as follows; inclusion criteria: 1. Age ≥ 18 years, 2. Patient had a clear diagnosis of non-valvular atrial fibrillation 3. The patient had signed the informed consent to admission and meets the ethical requirements. 4. Patient had underwent cardiac CT with complete period include the arterial enhancement phase, 1 and 3-min delay phase. 5. The patient underwent TEE and the transthoracic ultrasound examinations within 3 days of the CT examination and exclusion criteria: 1. Poor quality of cardiac CT scan, defined as 2 qualified experienced radiologist (>10 years working experience at cardiac CT diagnosis) judged that the image could not meet the diagnosis (expected to be caused by serious artifacts) or the CT value of left atrial enhancement phase was less than 200 HU, 2. Patients without the necessary clinical data or TEE images, and 3. Patients with a clinical diagnosis of non-thrombotic space occupying lesion, such as left atrial tumor. Figure 1 was the study flow chart for details of enrollment and exclusion. Three hundred and twenty-nine patients were enrolled in the study. The average age of the patients was 58.3 ± 12.2 (years ± years). 69.0% of the patients were male (accounting for% of the total), and the baseline risk factors and long-term labor types of the patients were additionally recorded. The baseline clinical data are shown in Table 1.

**TABLE 1:** Study characteristics of the of the overall populations.

Characteristics* patients (*n* = 329)	
Age^†^	58.3 ± 12.2
**Gender**	
Male	227 (69.0%)
Female	102 (31.0%)
BMI (kg/m2)^†^	25.7 ± 4.0
Hypertension	173 (52.6%)
Diabetes mellitus	42 (12.8%)
Hypercholesterolemia	135 (41.0%)
NYHA ≥ Level 3	42 (12.8%)
Peripheral vascular disease	43 (13.1%)
History of stroke/TIA	42 (12.8%)
**CHA2DS2-VAScscore**	
0	28 (8.5%)
1	51 (15.5%)
≥ 2	162 (76%)
**Type of atrial fibrillation**	
Paroxysmal atrial fibrillation	177 (53.8%)
Persistent atrial fibrillation	152 (46.2%)
Left atrial anteroposterior diameter (mm)^†‡^	39.0 ± 6.5

*Unless noted, all except age data are numbers of patients, with the percentage of patients in parentheses.*

*BMI, body mass index; TIA, Transient ischemic attack.

*^†^Age, BMI and the left atrial anteroposterior diameter were expressed as mean ± standard.*

*^‡^The left atrial anteroposterior diameter were measured at transthoracic ultrasound.*

### Computed Tomography Scanning Scheme of Left Atrium

All patients underwent 64-slice spiral CT scanning. Siemens SOMATOM sensing 64-slice spiral CT was used. All selected patients did not use β-receptor blockers to regulate heart rate. The imaging protocol includes a standard angiographic phase acquisition to evaluate the anatomical structure of the left atrium and pulmonary vein. Two delayed phase acquisitions 1 and 3 min after the injection of the contrast agent were used to distinguish between thrombosis (defined as a persistent defect with delayed acquisition of 3 min) and early filling artifacts. During the scanning, patients lay in the supine position, connected to the high-pressure syringe and electrode lead, and used the prospective electrocardiogram (ECG) gated scanning. Under the condition of single-breath holding, the ECG assisted scanning was performed. The prospective gated center is located at 75% of the RR interval, ranging from the aortic arch to the bottom of the heart. The tube voltage and current were adapted to the patient’s body mass index (BMI) (100 kVp for patients with BMI ≤ 30 kg/m^2^ and 120 kVp for patients with BMI > 30 kg/m^2^). The tube current automatic modulation CARE Dose4D technology was adopted and the rotation time was 350 ms. The slice thickness used in this study was 0.6 mm.

The contrast medium was a non-ionic isotonic contrast medium. The concentration of isopropanol was 350 mgI/ml injected at a speed of 60∼80/s. Then, approximately 30∼50 ml normal saline was injected at a speed of 4.5∼6 ml/s using bolus tracking automatic tracking technology. The region of interest was placed in the ascending aorta. When the CT value of the aorta rose to 100 Hu, the scanning was delayed for an additional 6 s. After reaching the threshold, the enhancement phase scanning was automatically performed. The scanning length, time, and dose-length product (DLP) of CT are recorded from the scanner console, and the DLP is multiplied by the previously recommended conversion factor (0.014 msv/cGy/cm) to calculate the effective radiation dose at different stages. All scans were performed by experienced technicians who have been engaged in cardiac CT scanning for more than 3 years and were carried out in strict accordance with the operation specifications. After obtaining the scans and preliminary processing, the supporting workstation was used to reconstruct the volume rendering images. Finally, the images were analyzed and the diagnosis made.

### Diagnosis of the Computed Tomography Image

**FIGURE 2:**
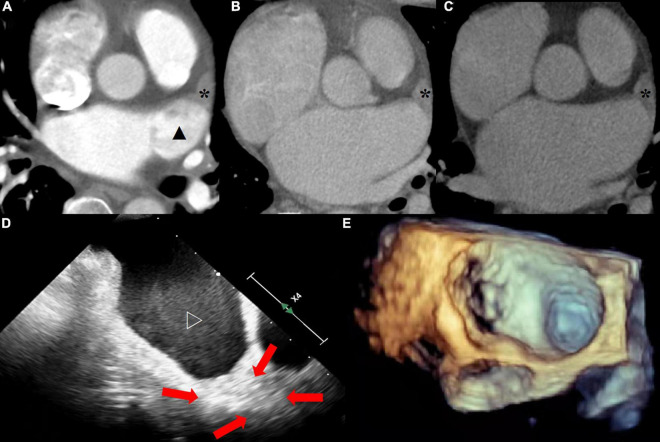
**(A)** The arterial enhanced phase shows the filling defect (solid star) at the tip of the left atrial appendage and the uneven filling (solid triangle) of contrast medium in the rest of the structure. **(B)** The tip of left atrial appendage still showed filling defect, while the rest were filled evenly at 1-min delayed. **(C)** Thrombus at the tip of the left atrial appendage was confirmed at 3-min delayed. **(D)** The tip of left atrial appendage was old thrombus (red arrow) and the rest of the structure were severe SEC (hollow triangle) at TEE. **(E)** Image of 3D ultrasound.

All CT images were independently evaluated by three experienced diagnostic doctors blind to each other, at the arterial enhancement phase (Y.Z a 3-year experiences in cardiovascular CT diagnosis), 1-min (J.W a 3-year experiences in cardiovascular CT diagnosis), and 3-min delay phase (X. L a 5-year experiences in cardiovascular CT diagnosis), respectively. Objective evaluation indexes included average CT value, noise, signal-to-noise ratio, and radiation dose of left atrial appendage area in each phase. The thrombus was evaluated by diagnosing the filling defect of left atrium observed in each enhancement phase (Figure 2).

### Inspection Methods and Diagnostic Criteria of Transesophageal Echocardiography

After anesthetizing the esophagus and throat with lidocaine, TEE was performed using Philips ie33 ultrasound. The working frequency of the probe was in the range 5.5–6 MHz. When the probe was 35 cm inside the esophagus, multiplane scanning was performed. After two-dimensional ultrasound, three-dimensional scanning was performed. The cardiac sections of two chambers and four chambers were detected through the middle section of the esophagus, in both the horizontal (0-degree) plane and the plane obtained by rotating the imaging sector from 0 to 180 degrees during continuous visualization of the LAA. The final judgment of thrombus was made by an experienced cardiac ultrasound physician (Q.W 10-year experience in cardiovascular ultrasound).

### Statistical Analysis

SPSS 22.0 statistical software and R language statistics package was used for the statistical analysis. A Kolmogorov-Smirnov test was used to check whether the data conforms to the normal distribution. The measurement data conforming to the normal distribution is represented by X ± *SD*, and the measurement data not conforming to the normal distribution is represented by M (Q1, Q3). A chi-square test was used to compare categorical variables. According to the binomial distribution and TEE as the reference standard, the differences between the diagnostic accuracy of the arterial enhancement phase, 1-min delay phase, and 3-min delay phase were tested. The diagnostic experimental indexes of different phases were evaluated using the 4-grid table method and the sensitivity, specificity, negative predictive value, and positive predictive value were calculated. Two correlated ROC curves comparison were used DeLong’s test. Multivariable logistic model was used for screening the risk factors which lead to a filling defect at 1-min delay phase so 3-min delay phase was still required. All potential confounders were entered into the model on the basis of known clinical relevance or significant association observed in univariate analysis. The odds ratio (OR) and 95% confidence interval (CI) of 1-min delay phase filling defect were computed. All statistical tests were two-sided and a *p*-value < 0.05 was considered statistically significant.

## Results

### Patient Characteristics for the Overall Populations

**FIGURE 3:**
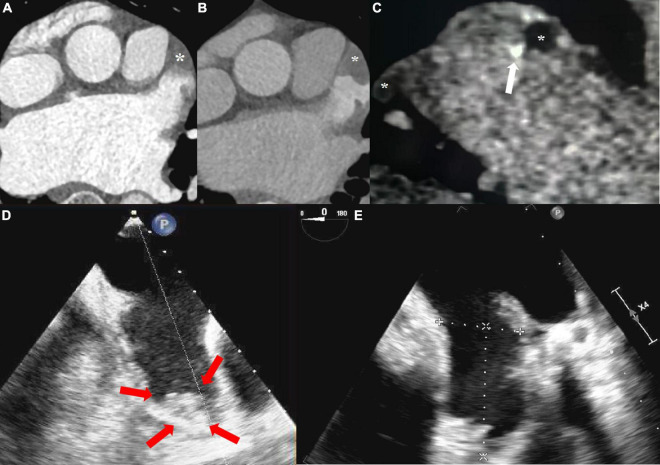
**(A)** The arterial enhanced phase shows the filling defect (solid star) at the tip and uneven filling in the body of the left atrial appendage. **(B)** Tip and body of left atrial appendage both showed filling defect at 1-min delayed. **(C)** Thrombus at the tip and body (mural and calcified marked with white arrow) of the left atrial appendage was confirmed at 3-min delayed. **(D)** The tip of left atrial appendage was fresh sludge thrombus (red arrow) at TEE in two chamber section. **(E)** After 6-weeks of regular oral anticoagulation treatment, re-examination of TEE showed no thrombus detected.

The age distribution of 329 patients was 58.3 ± 12.2 years, including 227 males (69.0%) and 102 females (31.0%). Among all patients with atrial fibrillation, paroxysmal atrial fibrillation accounted for 177 (53.8%) and persistent atrial fibrillation accounted for 152 (46.2%). Details of the baseline risk factors were shown in Table 1. Of 329 patients, 29 (8.8%) were diagnosed with thrombus and 95 (28.9%) spontaneous echocardiographic contrast at TEE (Figure 3).

### Diagnostic Accuracy of Different Enhanced Computed Tomography Phases

**FIGURE 4:**
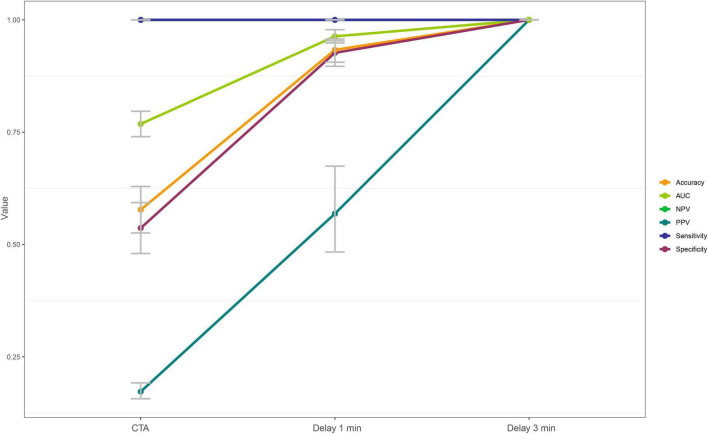
Different diagnostic efficiency of the arterial enhancement phase, 1-min delay phase, and 3-min delay phase for the left atrial thrombus detection.

A total of 173 patients (52.6%) had a filling defect in the arterial enhanced phase, of which 51 (29.5%) patients showed a filling defect after 1-min delayed, while 29 patients (56.9%) still had a filling defect in the 3-min delayed. Using the TEE of patients as the reference standard, For all phases, sensitivity and negative predictive were 100%. The specificity were 0.54, 0.93, and 1.00, respectively; The positive predictive values (PPV) were 0.17, 0.57, and 1.00, respectively; Area under curve (AUC) were 0.77, 0.96, and 1.00, respectively (Figure 4 and Table 2). There were significant statistical differences in the diagnostic accuracy of arterial enhanced phase, 1-min delayed scan and 3-min delayed (*p* < 0.0001 by DeLong’s test). A confusion matrix were shown in Table 3.

**TABLE 2:** Diagnostic Performance of the arterial enhanced phase, 1-min delayed and 3-min delayed phase for left atrial appendage thrombus detection.

	AUC	Accuracy	Sensitivity	Specificity	PPV	NPV
Arterial enhanced phase	0.77 (0.74–0.80)	0.58 (0.53–0.63)	1.00 (1.00–1.00)	0.54 (0.48–0.59)	0.17 (0.16–0.19)	1.00 (1.00–1.00)
1-min delayed phase	0.96 (0.95–0.98)	0.93 (0.91–0.96)	1.00 (1.00–1.00)	0.93 (0.90–0.95)	0.57 (0.48–0.67)	1.00 (1.00–1.00)
3-min delayed phase	1.00 (1.00–1.00)	1.00 (1.00–1.00)	1.00 (1.00–1.00)	1.00 (1.00–1.00)	1.00 (1.00–1.00)	1.00 (1.00–1.00)

*Data are expressed as value (%) and 95% confidence interval.*

*AUC, Area under curve; PPV, positive predictive value; NPV, negative predictive value.*

**TABLE 3:** Confusion matrix in the diagnosis of left atrial appendage thrombus by cardiac CT using TEE as the gold standard.

	Cardiac CT	Arterial enhancement phase		1-min delay phase		3-min delay phase	
		Thrombosis	No thrombosis	Thrombosis	No thrombosis	Thrombosis	No thrombosis
TEE	Thrombosis	29	0	29	0	29	0
	No thrombosis	139	161	22	278	0	300

### Logistic Regression Model for Screening Patients Who Could Avoided 1-min Scan

According to the proportion of filling defect in each phase, 29.5% of the patients who used 1-min delayed scanning still had filling defect, so 3-min delayed scanning was needed to diagnose thrombus. Therefore, we constructed a multiple logistic regression model to screen the high-risk factors of filling defect in 1-min delayed scanning. According to the results (definition of high risk factors, the odds ratios and 95% CIs for the baseline variables were shown in Table 4), reduced cardiac function (OR: 1.07, 95% CI 1.014–1.125, *p* = 0.01); increased CHA2DS2-VAScscore (OR: 4.88, 95% CI 2.672–8.905, *p* < 0.0001) and left atrial enlargement (OR: 1.07, 95% CI 1.014–1.125, *p* = 0.01) were independent risk factors of the 1-min delay filling defect at the multivariate analysis. Therefore, if patients with the above conditions were found filling defects enhanced in the arterial phase, they might be directly recommended for a 3-min delayed scan. In this study, if risk stratification was carried out according to this method, 88% (45 of 51) patients with 1-min delay filling defect would undergone 3-min delay scan directly, which saving 90 min at time and 76.7 msv at radiation dosage at total.

**TABLE 4:** Univariate and multivariate Logistic regression analysis of 1-min delay phase filling defect.

Risk factors	Logistic regression analysis					
	Univariate			multivariate		
	*P*-values	OR	95% CI	*P*-values	OR	95% CI
Reduced cardiac function	0.01	1.46	0.047–0.056	0.01	1.07	1.014–1.125
Increased CHA2DS2-VAScscore	< 0.0001	5.087	2.844–9.098	< 0.0001	4.88	2.672–8.905
Type of the atrial fibrillation	0.04	1.75	0.030–0.059	0.13	1.59	0.867–2.909
Left atrial enlargement	0.02	1.06	1.008–1.106	0.01	1.07	1.014–1.125

*All statistically significant baseline risk factors were calculated in logistic regression analysis, Reduced cardiac function classification were defined as NYHA ≥ Level 3; increased CHA2DS2-VAScscore were defined as male ≥ 2 or female ≥ 3; left atrial enlargement were defined as left atrial anteroposterior diameter ≥ 40 mm; OR, Odds Ratio; 95% CI, 95% confidence interval.*

### Scanning Dose and Enhanced Computed Tomography Image Quality

Average CT value of left atrial appendage was 368.3 ± 147.0 HU, 158.9 ± 87.1 HU and 109.0 ± 90.8 HU in the enhancement phase, 1 and 3-min delay, respectively. In the 3-min delay, the LA blood pool was 138.9 ± 36.4 HU, while the thrombus was 37.8 ± 9.8 HU, the ratio of the LA blood pool compared with the left atrial appendage was 3.7 ± 1.3 in thrombus and 1.1 ± 0.1 in normal (*p* < 0.0001). The average dose in the enhancement phase was 8.4 ± 7.8 msv (mean ± standard deviation). Compared with the enhancement phase, the increased radiation doses in the 1 and 3-min delay phases were 1.6 ± 1.3 msv and 1.9 ± 1.1 msv, respectively (Table 5).

**TABLE 5:** Scanning quality and the radiation dose.

Cardiac CT	Enhancement phase	1-min delayed	3-min delayed	Total
Average CT value of left atrial appendage (HU)	368.3 ± 147.0	158.9 ± 87.1	109.0 ± 90.8	NA
DLP (mGy•cm)	383.6 ± 326.7	125.9 ± 98.1	107.8 ± 58.1	520.4 ± 485.6
Effective dose (mSv)	5.3 ± 3.6	1.7 ± 1.3	1.5 ± 0.8	7.3 ± 6.8

*DLP, dose-length product.*

## Discussion

Cardiac CT is reliable in the diagnosis of left atrial appendage thrombus. However, it is not recommended by the guidelines owing to the heterogeneity of delayed scanning schemes. Our study explored the role of different early delayed phases in improving the diagnostic ability of cardiac CT for cardiogenic thrombus. We adopted the 1 and 3-min delayed phase technology implemented by most centers (21). Using TEE as the reference standard, it was calculated that the diagnostic efficiency has been significantly improved by 1 and 3 min delayed scanning (*p* < 0.0001), especially at specificity and PPV level.

The previous literature reported that the diagnostic efficiency of delayed cardiac scanning was similar to our research (21, 22), as compared with them, the sample size is currently known to be large, and the novelty of our research is the multi time point analysis but also provided a relatively reliable delay scanning scheme based on the predictors of the need for the 3-min scan. Hur et al. (17) preliminarily revealed the specificity of single-phase CT for the diagnosis of thrombus was to be 66.7%. Further research from the same team showed that the specificity of dual-phase CT for the diagnosis of thrombus was 93% (18). Spagnolo et al. (23) used three-phase delayed scanning to study the ability of delayed scanning to improve the accuracy of diagnosis of left atrial appendage thrombus. The results showed that delayed scanning could significantly improve the specificity and positive predictive value, which was similar to our results. However, their research focus was on the role of the 6-min late delay in improving the diagnostic accuracy.

The results of this study showed the importance of delayed scanning for the diagnosis of left atrial appendage thrombus. In patients with uneven left atrial enhancement in the arterial phase, thrombus cannot be directly diagnosed on CT images. Most patients (accounting for 84.5%) exhibit uneven filling due to delayed left atrial enhancement in the arterial phase. We hypothesize that patients with delayed left atrial arterial phase enhancement have hemodynamic abnormalities such as expansion of the left atrium, reduced ejection fraction, decreased blood flow rate, and increased afterload due to hypertension. In addition, the left atrium of patients with atrial fibrillation is enlarged and blood flow causes turbulence in the left atrial appendage. Therefore, when an arterial filling defect is found, it is necessary to perform delayed scanning. According to the study results, patients with reduced cardiac function, increased CHA2DS2-VAScscore and enlarged left atrial might be recommended for a 3-min delayed scan directly.

TEE is the current standard not only for thrombus evaluation in LAA but also for preprocedural planning prior to LAA exclusion in clinical practice. Compared with TEE, cardiac CT is a promising alternative to TEE that may improve device size selection for LAA occlusion, due to its acquisition of a three-dimensional dataset with high spatial resolution (24). However, cardiac CT scan has it’s limitations of iodine containing contrast agent allergy, damage to renal function and radiation, especially regarding the potential AKI. Patients requiring multiple cardioversions would be exposed to the cumulative radiation dose. On the other hand, those patients who cannot tolerate TEE process or have severe esophageal lesions such as active upper gastrointestinal bleeding and esophageal obstruction may benefit more from cardiac CT in comparison to the standard TEE. In addition to this, cardiac CT has the advantage of convenience for those who need to exclude LAA thrombus in emergency.

There were several limitations in our study. First, this study is a single-center retrospective study. As a confirmatory experiment to improve diagnostic accuracy, the retrospective research results are relatively reliable. However, multicenter and multiethnic research is still needed to improve regional and ethnic representation. Second, the delayed scan dose was larger than previous studies because the delayed scan range was not adjusted and reduced to the left atrial appendage. Moreover, this study proposed a high-risk stratification scheme to rationally reduce 1-min scanning, which hopefully could be further verified by randomized controlled studies in the future.

## Conclusion

Using TEE as the reference standard, early contrast-enhanced CT scanning with 1 and 3-min delay is necessary for the diagnosis of left atrium thrombus, which could significantly improve the diagnostic efficiency. Patients with high-risk stratification are suitable for direct 3-min delayed scanning.

## Data Availability Statement

The raw data supporting the conclusions of this article will be made available by the authors, without undue reservation.

## Ethics Statement

The studies involving human participants were reviewed and approved by the Ethics Committee of Fuwai Hospital, National Center for Cardiovascular Diseases. Written informed consent for participation was not required for this study in accordance with the national legislation and the institutional requirements. Written informed consent was not obtained from the individual(s) for the publication of any potentially identifiable images or data included in this article.

## Author Contributions

X-NL, J-XW, and BL contributed to conception, design, and administrative support. QW, X-YM, and Y-TZ collected and uploaded the data. X-NL and X-BY contributed to data analysis and interpretation. X-NL wrote the first draft of the manuscript. NZ and BL revised the article. All authors contributed to the article and approved.

## Conflict of Interest

X-BY was employed by Siemens Healthineers Ltd., and contributed to statistical analysis. Siemens Healthineers Ltd., conducted in the absence of any commercial or financial relationships in this research. Neither X-BY nor Siemens Healthineers Ltd., had control of the raw data. The remaining authors declare that the research was conducted in the absence of any commercial or financial relationships that could be construed as a potential conflict of interest.

## Publisher’s Note

All claims expressed in this article are solely those of the authors and do not necessarily represent those of their affiliated organizations, or those of the publisher, the editors and the reviewers. Any product that may be evaluated in this article, or claim that may be made by its manufacturer, is not guaranteed or endorsed by the publisher.

## Abbreviations

AKI: Acute kidney injury
AUC: Area under curve
AF: Atrial fibrillation
CT: Computed tomography
CI: Confidence Intervals
ECG: Electrocardiogram
NPV: Negative predictive value
OR: Odds ratios
PPV: Positive predictive values
ROC: Receiver operator characteristic curve
SD: Standard deviation
TEE: Transesophageal echocardiography.
